# Diseases of the Urinary Organs

**Published:** 1861-03

**Authors:** Lionel Beale

**Affiliations:** Physician to King’s College Hospital, &c.


### SELECTIONS.

*Diseases of the Urinary Organs. On the tests for Albumen in the Urine.* By Dr. Lionel Beale, F.R.S., Physician to King's College Hospital, &c.

The most important soluble substances present in the urine in disease, but absent in the healthy secretion, are *albumen*, *biliary constituents*, and *sugar*. The clinical importance of these is so great, that they deserve special attention.

*Albumen.*—The occurrence of albumen in the urine has been regarded as a most important pathological fact ever since the connection between dropsy and disease of the kidneys was established by Dr. Bright. Albumen, it need scarcely be said, is absent from the urine of healthy persons, although now and then it may be detected for a short period of time in the urine of individuals who are not suffering from any serious or permanent derangement of health. The presence of albumen must always be regarded by the physicians as a point of serious importance, although at the same time, *per se* it cannot be taken as evidence of the existence of any original lesion, unless it has been clearly detected from day to day for a certain time. Many of the cases which give rise to the escape of serum from the vessels in other parts of the body, independent of disease, will determine its transudation through the walls of the renal capillaries, and, as a matter of course, it will be found in the urine.

To recognise with certainty the presence of a substance in the urine having so important a bearing in the discovery and interpretation of certain morbid processes, as albumen, is obviously a point of the utmost importance to the practitioner. In the examination of the urine of patients, simple tests are at once applied, in order to determine if this substance be present or absent. In a majority of cases, such information is easily obtained; but occasionally an instance occurs in which, without great care, an erroneous conclusion is likely to be arrived at after the ordinary tests have been applied. As this question is one of very great practical importance, and of much interest, I propose to consider it at somewhat greater length than is usual in works devoted to the clinical examination of urine. The reactions to which I shall refer are not imaginary, but have actually occurred; and I have known instances in which albumen was stated to be absent when the urine contained a large quantity; and other specimens have fallen under my notice, which, although they really contained none, yielded a precipitate having many of the characters of albumen. Let us then investigate the phenomena *seriatim*.

1. *Albumen is usually precipitated from its solution upon the addition of a few drops of nitric acid.* Heller recommended that acid should be allowed to flow to the bottom of the tube containing the urine. In this manner, three strata are formed, the lowest stratum consisting of the pure acid, above which is the precipitated albumen, while the upper stratum consists of the fluid containing the albumen uncoagulated.

2. *Albumen is also generally coagulated by the application of heat* (140° to 167° Fahr.). If very dilute, a higher temperature is required. The best way of testing urine by heat is the following:—An ordinary test-tube is about half filled with the urine, and is to be held by the lower part. Heat is applied to a point near the *surface* of the fluid: the tube being shaken a little at the time, to prevent the glass being cracked. The slightest precipitate cannot fail to be observed, as the fluid below remains perfectly unchanged.—When urates are present, this plan is very useful, as we get three distinct strata; the upper one formed of coagulated albumen; the next clear, in consequence of the solution of the urates at a temperature somewhat below that necessary for the coagulation of the albumen; and lastly, the unchanged urates.

3. *If the solution of albumen be alkaline, no precipitate will be produced by heat. We are, therefore, generally directed to neutralise the alkali by an acid before heat is applied.* If excess of acid be added, the albumen is, of course, precipitated in the insoluble form, without the application of heat.

Frequently specimens of urine are met with which exhibit one or more of the following peculiarities, which tend either to make us believe that albumen is present when it is not, or cause us to conclude that it is absent when the urine contains it:

1. Upon the application of heat, the specimen may become turbid in consequence of the precipitation of phosphate. The reaction of the urine in this case would generally be neutral or feebly alkaline; but sometimes urine depositing phosphate on the application of heat is of a decidedly acid reaction.

2. Upon the addition of nitric acid, the specimen becomes turbid, in consequence of the decomposition of the urates held in solution in the urine, and the decomposition of uric in a granular state. If the acidified urine be boiled, it usually becomes clear with the development of a pinkish or brown color, consequent upon the decomposition of the uric acid.

3. Upon adding nitric acid to some specimens of urine of high specific gravity, an abundant precipitate of a crystalline character is produced. This consists of nitrate of urea, and is easily recognised by its crystalline character. It seldom appears immediately, and is hardly likely to be mistaken for albumen, except by a most superficial observer.

4. After adding a drop or two of nitric acid to urine suspected to contain albumen, in order to render it distinctly acid *no precipitate is produced upon boiling, although a large quantity of albumen may be present.* This is constant in all specimens of albumen urine, and shows the importance of never boiling urine suspected to contain albumen in a tube which may contain, by accident, a drop or two of nitric acid.

5. Cubebs, Copaiba, and some other resinous substances, taken internally, are said to give rise to precipitates in the urine which are liable to be mistaken for albumen.

*A Precipitate produced in Urine containing no Albumen. Phosphate resembling Albumen.* The precipitate of phosphates is very readily distinguished from albumen by its solubility in a little acid. Upon the addition of a few drops of nitric acid, the turbidity instantly disappears, and the solution becomes perfectly clear.

*Uric Acid resembling Albumen.*—When a precipitate of uric acid in a minute state of division is caused in consequence of the decomposition of the urates by nitric acid, its nature may be ascertained by allowing the mixture to stand for sometime, when the minute granules gradually increase in size, and at length become crystals, the nature of which is at once recognized upon microscopical examination. In some cases the crystal may be seen to form under the microscope. This precipitation of uric acid on adding nitric acid often leads to mistakes, and albumen is stated to be present in urine which really does not contain a trace. Several cases of this precipitate have occurred to myself, and I have heard of many others in the practice of friends. It has happened in the wards of our hospital, that the precipitate produced by nitric acid has been inferred by the clinical clerk to consist of albumen, when subsequently no precipitate could be obtained. The fallacy was explained by careful examination afterwards.

Dr. G. O. Rees has met with urine affording this precipitate of uric acid on the addition of nitric acid in cases of typhoid fever. Most of the instances which I have met with occurred in cases of liver affection. I extract two or three as examples.

*Urine from a Patient suffering from large Hydatid Tumours of the Liver.*—A small quantity of urine was filtered, and, upon the addition of a little nitric acid, a precipitate was produced. After standing a little while, this was examined by the microscope, and found to consist of minute crystals of uric acid. These were dissolved upon the application of heat; but, as the solution cooled, they were deposited again in the form of much larger crystals.

A specimen of urine exhibiting the same peculiarity contained excess of urea. Upon the addition of half its bulk of nitric acid, the mixture became nearly solid, from the formation of crystals of nitrate of urea. The deposit in this instance consisted partly of urate of soda.

Another example of which I have kept notes, occurred in a man aged 49, suffering from *rheumatic fever*. The urine was acid, specific gravity 1027, and containing much urate of soda. The practitioner who first saw the case boiled a portion of the urine. It remained clear; and he said, therefore, that it contained no albumen. A physician afterwards tested a portion of the same urine with nitric acid; and, finding that an abundant precipitate was produced, affirmed that much albumen was present. The deposit produced by nitric acid was found, by subsequent examination, to be dissolved by heat; and, when a portion was examined in the microscope, its true nature was decided by the presence of numerous uric acid crystals.

*No Precipitate produced in Urine containing Albumen.—Albumen not coagulated by heat when a little nitric acid is present.*—Upon the careful addition of a drop of nitric acid the precipitate at first formed when the acid comes in contact with the urine, slowly dissolves as it descends toward the bottom. Upon boiling this acidified solution, no precipitate of albumen will take place. Upon the further addition, however, of nitric acid, the albumen is precipitated.

This reaction has often led to mistakes. Not unfrequently albuminous urine has been poured into a test-tube which contained a trace of the nitric acid remaining from some previous experiment; and, upon boiling the mixture under these circumstances, no precipitate of albumen has occurred.

If a few drops of a dilute solution of nitric acid be added to a portion of albuminous urine in a test-tube, and the mixture boiled, no precipitate will take place. In fact, the addition of a dilute nitric acid will prevent the coagulation of albumen by heat. Dr. Bence Jones, was I believe, the first to explain this fact, in a communication to the editor of the 'Medical Gazette' (vol. xxvii, p. 289); and, so far as I can ascertain, no other explanation for the circumstance than that offered by him has been given. Dr. Jones thinks that the solution of albumen is owing to the formation of a nitrate of albumen which is soluble in a weak solution of nitric acid even although boiling, but insoluble in a mixture of acid of moderate strength. Dr. Bence Jones has also shown that albumen is not always precipitated from very acid urine upon the application of heat. From some experiments I have made, however, I have been led to conclude that the above result depends upon the decomposition of the phosphate by the nitric acid, and the consequent development of free phosphoric acid in which acid albumen is freely soluble. This view has been confirmed by some experiments which I made sometime since on the subject, and which have since been many times repeated. A weak solution of albumen was treated with a few drops of chloride of calcium, and afterwards with a little ammonia. After having stood for twenty-four hours, it was filtered. In this manner, any soluble phosphates present were removed. The solution was then tested as follows:

1. Albumen was precipitated by the application of heat, or by the addition of nitric acid, as usually occurs.

2. A very small quantity of dilute nitric acid did not prevent the coagulation of the albumen by heat.

3. After the addition of a few drops of phosphoric acid, the fluid no longer coagulated upon being boiled.

Some of the same solution as the above, which had not been treated with chloride of calcium and ammonia, afforded the same results upon the application of the tests as other albuminous solutions. A few drops of a weak solution of nitric acid, or a little phosphoric acid, prevented the precipitation of the albumen by heat. The addition of phosphoric acid to an albuminous solution, or a soluble phosphate and a little nitric acid, prevented the precipitation of the albumen by heat.

These results, therefore, led me to conclude that a trace of nitric acid prevents the coagulation of a moderately strong solution of albumen by heat, in consequence of decomposing the phosphates and setting free phosphoric acid, in which the albumen is soluble. When, however, excess of nitric acid is added, its action predominates over that of the phosphoric acid, and the albumen is precipitated.

At the same time, it must be admitted that there are several facts connected with the behaviour of weak solutions of albumen with acid, and under the influence of heat, which are not satisfactorily explained; and forms of albumen having different reactions are from time to time met with. The whole subject requires further careful investigation.

Scherer describes a variety of albumen which is only imperfectly coagulated by heat. It is probable, however, that many of the peculiar reactions met with from time to time depend upon the presence of other substances dissolved with the albumen, rather than upon any peculiar properties of the albumen itself, or the existence of a variety of this substance.

*Other Tests for Albumen.*—Albumen is precipited from its solutions by alcohol alum, and many metalic salts, as those of lead, mercury, copper, and silver. Bichloride of mercury is employed as a test, and ferrocyanide of potassium precipitates a solution of albumen to which acetic acid has been added. These salts will, however, produce precipitates in solutions of other substances allied to albumen.

*On Estimating the quantity of Albumen in Urine.*—The quantity of albumen varies much in different cases, sometimes amounting to a mere trace; while, in other instances, a proportion not much inferior to that present in serum has been met with. In order to estimate the quantity of albumen, it is only necessary to add acetic acid, and heat the urine in a water-bath to a temperature of 194° until it boils. The precipitate is to be collected on a weighed filter, well washed, dried, and weighed. The albumen always contains a small quantity of earthy salts, which are obtained by incineration. The residue must be deducted from the weight of the dried precipitate.

*Albuminous Urine*, from a patient with acute inflammation of the kidney. The deposit contained numerous granular casts, but no fat-cells were present; specific gravity, 1015; acid. The albumen coagulated by heat and nitric acid.

*New Substance allied to Albumen.*—Dr. Bence Jones obtained a new substance allied to albumen from the urine of a patient (under the care of Dr. Watson and Dr. MacIntyre) suffering from mollities ossium. The urine was slightly acid; specific gravity, 1034·2 (*Phil. Trans.* for 1848, p. 55). The deposit consisted of phosphate of lime, and cylinders of fibrine. Phosphates were precipitated by heat; but the urine was cleared by adding a drop of acid. No precipitate was produced by nitric acid; but, after being heated and left to cool, it became solid. The solid material was redissolved by heat, and precipitated again when the mixture became cool. On some days, the urine coagulated by boiling; on others, prolonged boiling produced no change. A specimen, which did not coagulate by boiling, was carefully examined. It was acid; specific gravity, 1039·6. It contained much urate of ammonia, phosphate of lime, and oxalate of lime.

The new substance was precipitated from the urine by alcohol, well washed, and ultimate analyses were made. It contained 1·09 per cent. of sulphur, and ·20 per cent. of phosphorus. This substance is the *hydrated deutoxide of albumen.* It was soluble in boiling water, and the precipitate produced by nitric acid was redissolved by heat, and it formed again as the mixture cooled. A similar substance occurs in small quantity in pus, and in the secretion from the vesiculæ seminales. The urine contained 66·97 parts per

1000 of this substance—an amount equal to the quantity of albumen in the blood. The patient was passing about 35 ounces of urine daily, which would contain upwards of 1000 grains, or more than two ounces of this new material.

Dr. Bence Jones recommends that this substance should be looked for again in acute cases of mollities ossium. He suggests that the reddening of the urine upon the addition of nitric acid might lead to its detection.—*Brit. Med. Journal, April* 21. 1860, *p.* 297.

